# Impact of Fellowship Training Type on Complication Rates in Primary and Revision Total Knee Arthroplasty by Recently Trained Board-Eligible Orthopedic Surgeons

**DOI:** 10.7759/cureus.85637

**Published:** 2025-06-09

**Authors:** Allyson N Pfeil, Joshua H Taylor, Purvi Desai, Corey F Hryc, Warren R Dunn, Anay R Patel

**Affiliations:** 1 Research, Fondren Orthopedic Research Institute, Houston, USA; 2 Engineering Medicine, Texas A&M School of Engineering Medicine, Houston, USA

**Keywords:** abos, american board of orthopaedic surgery, complications, early-career surgeons, fellowship, orthopaedic subspecialization, revision tka, specialty, surgical complications, total knee arthroplasty

## Abstract

Background: Demand for primary total knee arthroplasty (TKA) and revision TKA (rTKA) procedures is projected to increase. Many orthopedic surgery residents pursue fellowship training following residency. In investigating the impact of fellowship subspecialization on surgical outcomes, we hypothesize that procedures performed by arthroplasty-trained orthopedic surgeons will have the most favorable complication, readmission, and reoperation rates compared to other fellowships or those without fellowship training.

Materials and methods: The American Board of Orthopaedic Surgeons (ABOS) database was queried for all adult cases of TKA and rTKA submitted between 2003 and 2019, identifying 64,437 cases performed by 4,758 candidates. Fellowship groupings included adult reconstruction (AR), sports medicine (SM), other (OTH), and no fellowship (NO). Adverse events, including complications, readmissions, and reoperations, were collected for each case. Multivariate logistic regression analyses were used in congruence with the Wald test. Chi-square tests assessed descriptive data. Statistically significant P-values were defined at P < 0.05.

Results: NO reported the least surgical and medical complications at 10.25% (n=1176) and 7.25% (n=832), respectively (P=0.001). Of AR cases, 11.6% (n=4652) experienced surgical complications. AR achieved the lowest reoperation rate of 4.45% (n=965) (P=0.002). Cases managed by SM incurred a 20% decreased readmission risk (P=0.032) compared to AR.

Conclusions: AR candidates reported the lowest rates of reoperation and comparatively low rates of surgical complications. However, NO generalists reported the lowest rate of surgical and medical complications, which may be influenced by patient selection.

## Introduction

Total knee arthroplasty (TKA) is a safe and effective procedure addressing osteoarthritis and other pathologies of the knee [1]. Primary TKA utilization is increasing dramatically [2-4], and in the United States alone, TKAs are expected to increase 143% by 2050 [5]. As TKA volume rises, a corresponding increase in additional surgical interventions, known as a revision TKA (rTKA), is anticipated. Consequently, the incidence of rTKA is projected to grow between 78% and 182% by 2030 [6].

To address heightened surgical demand, more orthopedic residents are pursuing fellowship training to subspecialize [7,8]. Outcomes-based clinical research studies have reported that patients of fellowship-trained surgeons benefit from superior clinical outcomes compared to those treated by surgeons without auxiliary training [7-10]. Despite these positive findings, no study to our knowledge has verified the impact of fellowship training on TKA and rTKA outcomes utilizing a national database of early-career surgeons.

Based on this shortcoming, our objective was to compare rates of adverse events in TKA and rTKA cases managed by board-eligible surgeons applying for the American Board of Orthopaedic Surgery (ABOS) Part II oral examination, the final step in attaining ABOS board certification. These rates would be compared across subspecialties, including adult reconstruction (AR), sports medicine (SM), other fellowships (OTH), and no fellowship (NO). Congruent with prior research [7-10], we hypothesized that arthroplasty-trained, early-career orthopedic surgeons would have superior complication, readmission, and reoperation rates compared to those with OTH or NO.

## Materials and methods

The study was conducted at the Fondren Orthopedic Research Institute, located within the Texas Orthopedic Hospital, Houston, Texas. ABOS granted permission to all knee arthroplasty case data submitted by ABOS Part II oral examination candidates between 2002 and 2018 [11]. The accessed cases had been submitted by candidates during a six-month surgical case collection window, coinciding with the ABOS collection period, from 2003 to 2019. These ABOS board eligible candidates were within their first four years of practice.

Following institutional review board (IRB) approval, the group queried all primary and rTKAs identified by current procedural terminology (CPT) codes 27447 and 27487, respectively, from the ABOS database. Patients under 18 years of age were omitted, resulting in 120 exclusions. The final cohort comprised 64,437 cases, consisting of 59,108 TKAs and 5,329 rTKAs, submitted by 4,758 candidates.

Collected variables included patient demographics (age, sex), candidate fellowship type, CPT code, adverse events (surgical and medical complications defined by ABOS, readmission, reoperation) within 90 days of surgery, and length of follow-up. Of note, readmission and reoperation data were not collected by ABOS prior to 2013. Fellowship classifications were defined as follows: AR, SM, OTH, and NO. AR candidates graduated from an AR fellowship with or without an additional fellowship. SM candidates completed a sports medicine fellowship with or without other fellowships, excluding AR. OTH candidates reported finishing any fellowship, excluding AR and SM, such as pediatrics, oncology, trauma, and shoulder, among others. NO candidates did not report attending a fellowship program.

Statistical analysis

Multivariable logistic regression analyses computed coefficients, risk ratios, and confidence intervals, and the Wald test elicited P-values from the coefficients. For all regression analyses, male sex and AR fellowship served as reference groups to determine relative comparative association. Chi-square tests evaluated descriptive data. Statistically significant P-values were defined at P < 0.05; confidence intervals were calculated at 95%. All statistical analyses were computed using either Python v3.12 (Python Software Foundation, Wilmington, Delaware) or GraphPad (GraphPad Software, Boston, Massachusetts).

## Results

Analyses from the candidate and case data

The 64,437 cases were stratified as 40,110 AR, 8,652 SM, 4,207 OTH, and 11,468 NO (Table 1).

**Table 1:** Case characteristics by fellowship type. AR: adult reconstruction; sports: sports medicine; TKA: total knee arthroplasty; rTKA: revision total knee arthroplasty

Features	AR (%)	SM (%)	Other (%)	None (%)	Total
Candidates	1346 (28.29)	1530 (32.16)	676 (14.21)	1206 (25.35)	4758
Cases	40110 (62.25)	8652 (13.43)	4207 (6.53)	11468 (17.80)	64437
Cases per candidate	29.80	5.65	6.22	9.51	13.54
TKA cases	35764 (60.51)	8511 (14.40)	3882 (6.56)	10951 (18.53)	59108
rTKA cases	4346 (81.56)	141 (2.65)	325 (6.10)	517 (9.70)	5329
Females (cases)	24976 (62.27)	5451 (63.00)	2513 (59.73)	7154 (62.38)	40094 (62.22)
Age (cases)	65.58 ± 10.24	66.40 ± 9.74	65.51 ± 10.90	66.01 ± 10.23	65.76 ± 10.22
Follow-up (weeks)	8.86 ± 5.94	9.36 ± 6.29	9.12 ± 6.44	8.80 ± 6.31	8.93 ± 6.09

**Figure 1:**
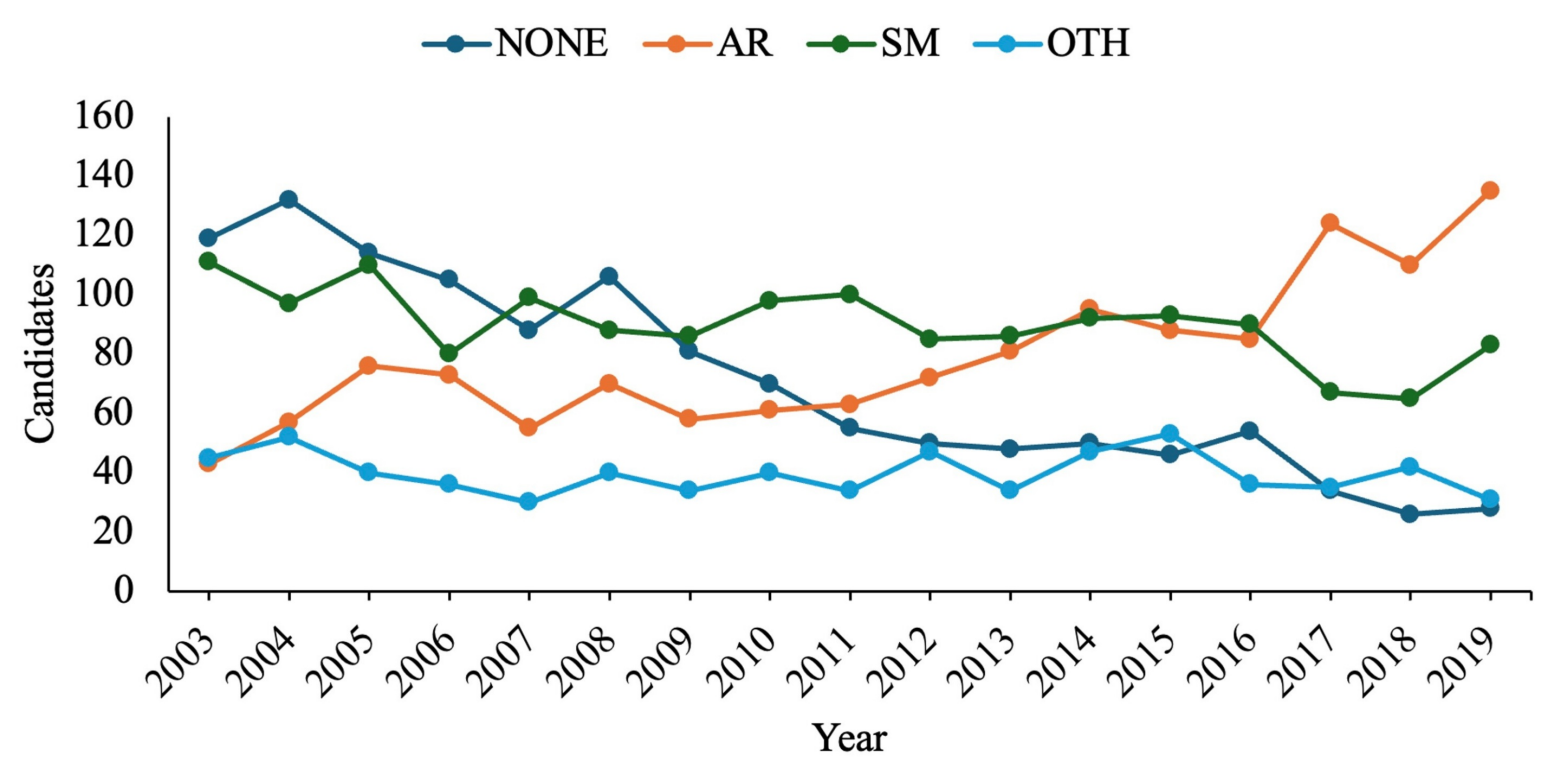
Fellowship utilization trends by candidate. AR: adult reconstruction; SM: sports medicine; OTH: other

**Figure 2:**
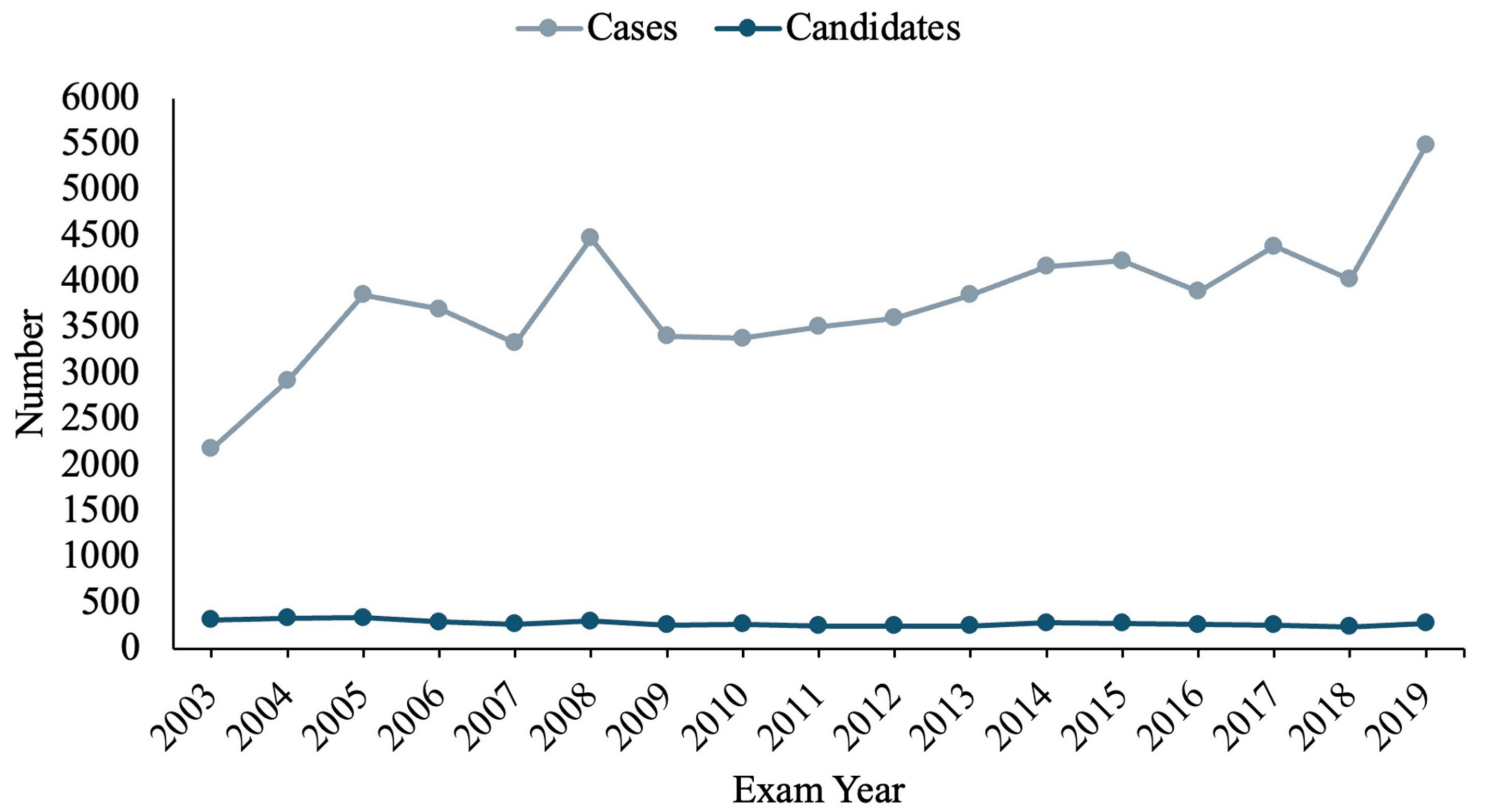
Trends in included cases and candidates attempting ABOS part II exam. ABOS: American Board of Orthopaedic Surgeons

The cases consisted of 62.2% females, averaging 65.8 years old, with an 8.9-week follow-up. These cases were under the care of 4,758 candidates; fellowship utilization, as well as case per candidate trends over time, can be found in Figures 1, 2.

**Figure 3:**
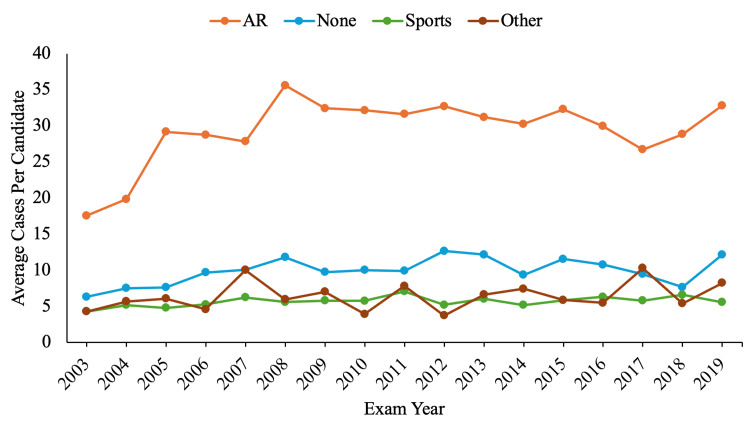
Trends in included average cases per candidate by fellowship type attempting ABOS part II exam. AR: adult reconstruction; SM: sports medicine; ABOS: American Board of Orthopaedic Surgeons

The average number of cases per candidate was calculated and examined yearly (Figure 3).

AR performed the most procedures at 29.8 cases per candidate during the 17-year window.

Surgical and medical complications

Overall, 11.6% of cases incurred surgical complications, 8.9% medical complications, 4.6% reoperations, and 5.3% readmissions (Table 2).

**Table 2:** Complication characteristics by fellowship type and overall complication rates by fellowship. P-values calculated using the chi-square test. Bold P-values denote statistical significance. AR: adult reconstruction; sports: sports medicine

Complications	AR (%)	SM (%)	Other (%)	None (%)	P-value
Cases from 2002-2018	40110	8652	4207	11468	
Surgical (n=7499)	4652 (11.60)	1125 (13.00)	546 (12.98)	1176 (10.25)	0.001
Medical (n=5754)	3741 (9.32)	772 (8.92)	409 (9.72)	832 (7.25)	0.001
Cases from 2013-2018	21701	3393	1930	3028	
Reoperation (n=1387)	965 (4.45)	161 (4.75)	122 (6.32)	139 (4.59)	0.002
Readmission (n=1589)	1165 (5.37)	165 (4.86)	115 (5.96)	144 (4.76)	0.188

A breakdown of the top 10 medical and surgical complication types is summarized in Table 3.

**Table 3:** Most frequently occurring surgical and medical complications. Counts reflect the total of the most commonly occurring (and listed) surgical and medical complications. The overall total counts can be found in Table 2.

Complication	Number (% frequency of all cases)	Proportion of all surgical or medical complications
Surgical complication	n=7081	
Bone fracture	630 (0.98)	7.51
Infection	1271 (1.97)	15.14
Nerve palsy/injury	334 (0.52)	3.98
Skin ulcer/blister	649 (1.01)	7.73
Stiffness/arthrofibrosis	1053 (1.63)	12.54
Surgical procedure intervention	251 (0.39)	2.99
Surgical unspecified	1587 (2.46)	18.91
Tendon/ligament injury	308 (0.48)	3.67
Wound dehiscence	359 (0.56)	4.28
Wound healing delay/failure	639 (0.99)	7.61
Medical complications	n=5665	
Anemia	1095 (1.70)	15.64
Arrythmia	214 (0.33)	3.06
Confusion/delirium	292 (0.45)	4.17
Deep venous thrombosis	287 (0.45)	4.10
Medical unspecified	2339 (3.63)	33.41
Pneumonia	203 (0.32)	2.90
Pulmonary embolism	377 (0.59)	5.38
Renal failure	349 (0.54)	4.99
Urinary retention	291 (0.45)	4.16
Urinary tract infection	218 (0.34)	3.11

NO reported the least surgical complications at 10.3%, followed by AR at 11.6%, and OTH and SM at 13.0% each (P=0.001) (Table 2). Cases by SM and OTH incurred a greater risk of surgical complications (+26 and +16%, respectively; P < 0.001) (Tables 4, 5).

**Table 4:** Multivariate logistic regression results of risk factors for surgical and medical complications. P-values calculated using the Wald test. Bold P-values designate statistical significance. CI: confidence interval; TKA: total knee arthroplasty; AR: adult reconstruction; sports: sports medicine

Variable	Risk ratio (CI)	P-value
Surgical complication		
Age	0.86 (0.84 - 0.88)	< 0.001
Female sex	1.00 (0.91 - 1.00)	0.072
Medical complication	1.88 (1.74 - 2.02)	< 0.001
Revision TKA	2.08 (1.93 - 2.23)	< 0.001
AR	1.0 – reference	-
Sports	1.26 (1.18 - 1.36)	< 0.001
None	0.94 (0.88 - 1.01)	0.095
Other	1.16 (1.06 - 1.28)	0.002
Intercept	0.11 (0.11 - 0.12)	< 0.001
Medical complication		
Age	1.41 (1.37 - 1.45)	< 0.001
Female sex	0.86 (0.82 - 0.91)	< 0.001
Surgical complication	1.86 (1.73 - 2.01)	< 0.001
Revision TKA	1.40 (1.28 - 1.53)	< 0.001
AR	1.0 – reference	-
Sports	0.95 (0.88 - 1.04)	0.274
None	0.77 (0.72 - 0.84)	< 0.001
Other	1.04 (0.94 - 1.16)	0.449
Intercept	0.09 (0.09 - 0.10)	< 0.001

**Table 5:** Rates of adverse events by fellowship type and procedure. TKA: total knee arthroplasty; rTKA: revision total knee arthroplasty; AR: adult reconstruction; SM: sports medicine; OTH: other; NO: none

Variable	AR	SM	OTH	NO
Primary TKA				
Surg complication	3754 (10.50)	1096 (12.88)	467 (12.03)	1106 (10.10)
Med complication	3165 (8.85)	762 (8.95)	352 (9.07)	796 (7.27)
Readmission	943 (4.89)	162 (4.86)	96 (5.52)	134 (4.60)
Reoperation	785 (4.07)	158 (4.74)	97 (5.57)	126 (4.32)
Revision TKA				
Surg complication	898 (20.66)	29 (20.57)	79 (24.31)	70 (13.54)
Med complication	576 (13.25)	10 (7.09)	57 (17.54)	36 (6.96)
Readmission	222 (9.21)	3 (4.84)	19 (10.00)	10 (8.77)
Reoperation	180 (7.47)	3 (4.84)	25 (13.16)	13 (11.40)

Medical complications and revisions were positively correlated with surgical complications (+88 and +108%, respectively; P < 0.001). Regarding medical complications, NO reported the least at 7.3%, corresponding to a 23% decreased risk of occurrence (P=0.001) (Table 4). Medical complications were statistically influenced by revisions and surgical complications (P < 0.001).

Reoperation and readmission

Reoperation rates varied among the four fellowship types; AR reported the lowest rate at 4.5%, contrasting with OTH at the highest, with 6.3% (P=0.002) (Table 2). Additionally, cases by OTH experienced an elevated reoperation risk at 46% (P=0.004) (Table 6).

**Table 6:** Logistic regression results of reoperation and readmissions for included cases from 2013-2018. P-values calculated using the Wald test. Bold P-values designate statistical significance. CI: confidence interval; TKA: total knee arthroplasty; AR: adult reconstruction; sports: sports medicine; NA: not available

Variable	Risk ratio (CI)	P-value
Reoperation		
Age	0.75 (0.70 - 0.80)	< 0.001
Female sex	1.04 (0.90 - 1.20)	0.607
Medical complication	0.49 (0.40 - 0.60)	< 0.001
Surgical complication	30.85 (26.37 - 36.09)	< 0.001
Revision	1.03 (0.84 - 1.27)	0.774
Readmission	25.58 (21.36 - 30.64)	< 0.001
AR	1.0 – reference	-
Sports	1.05 (0.84 - 1.31)	0.663
None	1.10 (0.86 - 1.40)	0.459
Other	1.46 (1.13 - 1.89)	0.004
Intercept	0.01 (0.00 - 0.01)	< 0.001
Readmission		
Age	1.15 (1.08 - 1.22)	< 0.001
Female sex	0.76 (0.67 - 0.86)	< 0.001
Medical complication	10.33 (10.33 - 13.59)	< 0.001
Surgical complication	2.19 (1.87 - 2.55)	< 0.001
Revision	1.21 (1.02 - 1.45)	< 0.001
Reoperation	32.89 (27.41 - 39.47)	< 0.001
AR	1.0 – reference	-
Sports	0.80 (0.66 - 0.98)	0.032
None	0.89 (0.72 - 1.10)	0.292
Other	0.83 (0.65 - 1.06)	0.143
Intercept	0.02 (0.01 - 0.02)	< 0.001

Surgical complications and readmissions corresponded to a 30.9 and 25.6x reoperation probability, respectively, while medical complications decreased reoperation risk by 51% (P < 0.001) (Table 6). Interestingly, SM cases experienced a 20% reduction in readmission risk (P=0.032). Revision, reoperation, surgical, and medical complications all increased readmission risk (P < 0.001).

## Discussion

This study sought to evaluate variations in clinical outcomes following TKAs and rTKAs performed by ABOS Part II oral examination candidates stratified by fellowship type. AR candidates reported the lowest reoperation rate; however, this did not extend to the other variables under consideration. Thus, the findings only partially support the initial hypothesis of AR superiority with respect to clinical outcomes. This is incongruent with prior literature, which, although lacking in geographical generalizability, described a relationship between fellowship-trained surgeons and excellent TKA outcomes [7,12]. However, reoperation might be considered the most significant variable studied, which would corroborate prior research findings.

Although AR candidates documented a low surgical complication rate, NO achieved the lowest. Interestingly, NO also reported the lowest medical complication rate, possibly indicating that NO candidates managed cases with the least medical complexity. Consequently, candidates with a fellowship, often referred to as specialists, provided care to 85.5% of all cases with medical complications. The positive correlation between surgical and medical complications may allude to patient selection and attributes influencing the superior complication rate reported by NO.

No substantial determination could be made regarding AR affecting the highest rates of readmission. Again, considering patient selection, AR patients with high rates of medical complexity may have necessitated a return to the hospital for indications other than surgery. Additionally, rTKAs were highly overrepresented by AR candidates and likewise associated with higher readmissions.

National database study results defined short-term, primary TKA readmission and reoperation rates at 3.5% and 1.2%, respectively [13]. This contrasts with the averages of ABOS Part II candidates at 4.9% and 4.3%, respectively. The same database described short-term rTKA reoperations at 3.9% [14], incongruent with the candidate rate of 8.0%. Observed differences between the literature and candidate rates seemingly suggest further proficiency is achieved or gained following the board-eligible period.

Both the literature and the study results describe more orthopedic residents subspecializing with fellowship training (Figure 1) [15,16]. This uptick has been attributed to the progressively competitive surgical landscape [17-19]. Additionally, labor market trends may influence fellowship utilization, given that more jobs continually prefer or require subspecialization within orthopedic surgery [20,21].

Prior studies have utilized ABOS Part II oral examination case list data to analyze the impact of fellowship on surgical outcomes extraneous to joint arthroplasty. These studies found substantive differences among fellowship types. In clavicle fractures, researchers discovered that trauma fellows reported the lowest rate of surgical complications [10]. Similarly, a study on total shoulder arthroplasty found lower surgical complications in the shoulder fellowship cohort [8].

The causality of fellowship type impacting clinical outcomes is sometimes attributed to surgical volume [22-24]. The volume-outcome relationship theoretically arises from either repetitive practice or a favorable reputation preluding outcomes [25]. This occurrence could explain the higher reoperation and surgical complication rates reported by OTH and SM. Since SM, OTH, and NO performed only six to ten cases per candidate compared to AR with thirty, the lower-volume surgeons would be predicted to have less favorable TKA outcomes. However, this fails to account for NO besting AR in surgical complications.

Limitations

There are several limitations to this study. To begin, all data is self-reported by candidates, which may result in discrepancies. Candidates often reported various complications as undefined, which obscured the analysis of specific complication rates. Readmission and reoperation rates were not recorded until 2013 and may be dissimilar to years prior. Moreover, the large study window (2003-2019) may introduce variability due to evolving surgical practices. The ABOS dataset did not include clinical details to assess individual case complexity, such as comorbidities, American Society of Anesthesiologists (ASA) class, BMI, or hospital setting, which limits our ability to control for potential selection bias. Additionally, the reasons for readmission and reoperation were undisclosed, preventing the analysis of reasons in the study. Auxiliary data regarding the nature of readmission and reoperation were unavailable for analysis. Follow-up was also limited to an average of 8.9 weeks.

Note: We did not include case volume in the regression due to its strong overlap with fellowship type, which would introduce multicollinearity and reduce model interpretability. This limitation should be considered when interpreting differences in complication, readmission, and reoperation rates across fellowship types.

## Conclusions

AR candidates reported the lowest rates of reoperations and comparatively low rates of surgical complications. Additionally, AR candidates assumed responsibility for a substantial number of cases with medical complications and more complex rTKAs. As the demand for TKA and rTKA rises, fellowship-trained surgeons will form the vanguard of treatment for an increasingly complex patient population. 

## References

[1] Mathis DT, Lohrer L, Amsler F, Hirschmann MT (2021). Reasons for failure in primary total knee arthroplasty - an analysis of prospectively collected registry data. J Orthop.

[2] Nham FH, Patel I, Zalikha AK, El-Othmani MM (2023). Epidemiology of primary and revision total knee arthroplasty: analysis of demographics, comorbidities and outcomes from the national inpatient sample. Arthroplasty.

[3] Shichman I, Roof M, Askew N, Nherera L, Rozell JC, Seyler TM, Schwarzkopf R (2023). Projections and epidemiology of primary hip and knee arthroplasty in Medicare patients to 2040-2060. JB JS Open Access.

[4] Klug A, Gramlich Y, Rudert M, Drees P, Hoffmann R, Weißenberger M, Kutzner KP (2021). The projected volume of primary and revision total knee arthroplasty will place an immense burden on future health care systems over the next 30 years. Knee Surg Sports Traumatol Arthrosc.

[5] Inacio MC, Paxton EW, Graves SE, Namba RS, Nemes S (2017). Projected increase in total knee arthroplasty in the United States - an alternative projection model. Osteoarthritis Cartilage.

[6] Schwartz AM, Farley KX, Guild GN, Bradbury TL Jr (2020). Projections and epidemiology of revision hip and knee arthroplasty in the United States to 2030. J Arthroplasty.

[7] Mahure SA, Feng JE, Schwarzkopf RM, Long WJ (2020). The impact of arthroplasty fellowship training on total joint arthroplasty: comparison of peri-operative metrics between fellowship-trained surgeons and non-fellowship-trained surgeons. J Arthroplasty.

[8] Gombera MM, Laughlin MS, Vidal EA, Brown BS, Morris BJ, Edwards TB, Elkousy HA (2020). The impact of fellowship type on trends and complications following total shoulder arthroplasty for osteoarthrosis by recently trained board-eligible orthopedic surgeons. J Shoulder Elbow Surg.

[9] Mabry SE, Cichos KH, McMurtrie JT, Pearson JM, McGwin G Jr, Ghanem ES (2019). Does surgeon fellowship training influence outcomes in hemiarthroplasty for femoral neck fracture?. J Arthroplasty.

[10] Gombera MM, Morris BJ, Elkousy HA, Laughlin MS, Vidal EA, Brinker MR (2021). Trauma fellowship impact on trends and complications of operatively treated clavicle fractures in recently trained orthopedic surgeons. J Clin Orthop Trauma.

[11] (2003-2019). ABOS data for research. http://www.abos.org/research/abos-data-for-research/.

[12] Singh V, Simcox T, Aggarwal VK, Schwarzkopf R, Long WJ (2021). Comparative analysis of total knee arthroplasty outcomes between arthroplasty and nonarthroplasty fellowship trained surgeons. Arthroplast Today.

[13] George J, Chughtai M, Khlopas A, Klika AK, Barsoum WK, Higuera CA, Mont MA (2018). Readmission, reoperation, and complications: total hip vs total knee arthroplasty. J Arthroplasty.

[14] Dieterich JD, Fields AC, Moucha CS (2014). Short term outcomes of revision total knee arthroplasty. J Arthroplasty.

[15] Horst PK, Choo K, Bharucha N, Vail TP (2015). Graduates of orthopaedic residency training are increasingly subspecialized: a review of the American Board of Orthopaedic Surgery part II database. J Bone Joint Surg Am.

[16] Daniels AH, DiGiovanni CW (2014). Is subspecialty fellowship training emerging as a necessary component of contemporary orthopaedic surgery education?. J Grad Med Educ.

[17] Egro FM, Smith BT, Murphy CP, Stavros AG, Nguyen VT (2021). The impact of fellowship training in academic plastic surgery. Ann Plast Surg.

[18] DeFroda SF, Shah KN, Safdar O, Mulcahey MK (2018). Trends in research productivity of residents applying for orthopedic sports medicine fellowship. Phys Sportsmed.

[19] Borman KR, Vick LR, Biester TW, Mitchell ME (2008). Changing demographics of residents choosing fellowships: longterm data from the American Board of Surgery. J Am Coll Surg.

[20] Morrell NT, Mercer DM, Moneim MS (2012). Trends in the orthopedic job market and the importance of fellowship subspecialty training. Orthopedics.

[21] Mannava S, Jinnah AH, Cinque ME (2018). An analysis of Orthopaedic job trends in the United States over the past 30 years. J Am Acad Orthop Surg Glob Res Rev.

[22] Bini S, Khatod M, Cafri G, Chen Y, Paxton EW (2013). Surgeon, implant, and patient variables may explain variability in early revision rates reported for unicompartmental arthroplasty. J Bone Joint Surg Am.

[23] Lau RL, Perruccio AV, Gandhi R, Mahomed NN (2012). The role of surgeon volume on patient outcome in total knee arthroplasty: a systematic review of the literature. BMC Musculoskelet Disord.

[24] Kazarian GS, Lawrie CM, Barrack TN, Donaldson MJ, Miller GM, Haddad FS, Barrack RL (2019). The impact of surgeon volume and training status on implant alignment in total knee arthroplasty. J Bone Joint Surg Am.

[25] Luft HS, Hunt SS, Maerki SC (1987). The volume-outcome relationship: practice-makes-perfect or selective-referral patterns?. Health Serv Res.

